# Hypoxia‐induced YAP activation and focal adhesion turnover to promote cell migration in mesenchymal TNBC cells

**DOI:** 10.1002/cam4.5680

**Published:** 2023-02-09

**Authors:** Thi My Hang Nguyen, Yi‐Shyun Lai, Ying‐Chi Chen, Tzu‐Chien Lin, Ngoc Thang Nguyen, Wen‐Tai Chiu

**Affiliations:** ^1^ Department of Biomedical Engineering, College of Engineering National Cheng Kung University Tainan Taiwan; ^2^ Department of Chemistry National Cheng Kung University Taiwan Taiwan; ^3^ Institute of Basic Medical Sciences, College of Medicine National Cheng Kung University Tainan Taiwan; ^4^ Medical Device Innovation Center National Cheng Kung University Tainan Taiwan

**Keywords:** cell migration, focal adhesions, hypoxia, mesenchymal TNBC, YAP

## Abstract

**Background:**

Hypoxia is commonly characterized by malignant tumors that promote the aggressiveness and metastatic potential of cancer. Triple‐negative breast cancer (TNBC) is the most aggressive subtype of breast cancer, with approximately 46% capacity related to distant metastasis. Transcriptional factor yes‐associated protein (YAP), a core component of the Hippo pathway, is associated with poor prognosis and outcome in cancer metastasis. Here, we explored the effect of hypoxia‐mediated YAP activation and focal adhesions (FAs) turnover in mesenchymal TNBC cell migration.

**Methods:**

We characterized the effect of hypoxia on YAP in different breast cancer cell lines using a hypoxia chamber and CoCl_2_.

**Results:**

Hypoxia‐induced YAP nuclear translocation is significantly observed in normal breast epithelial cells, non‐TNBC cells, mesenchymal TNBC cells, but not in basal‐like TNBC cells. Functionally, we demonstrated that YAP activation was required for hypoxia to promote mesenchymal TNBC cell migration. Furthermore, hypoxia induced the localization of FAs at the leading edge of mesenchymal TNBC cells. In contrast, verteporfin (VP), a YAP inhibitor, significantly reduced the migration and the recruitment of nascent FAs at the cell periphery under hypoxia conditions, which only showed in mesenchymal TNBC cells.

**Conclusions:**

Our data support the hypothesis that YAP is novel factor and positively responsible for hypoxia‐promoting mesenchymal TNBC cell migration. Our findings provide further evidence and outcomes to help prevent the progression of TNBC.

## Graphical abstract

Identifying YAP as a key mediator of hypoxia‐induced mesenchymal TNBC cell migration based on the following evidence. (1) Hypoxia triggers YAP nuclear translocation at high cell density in mesenchymal TNBC; (2) Hypoxia only promotes mesenchymal TNBC cell migration, and YAP plays a crucial role in cell migration under hypoxia; and (3) Hypoxia promotes focal adhesions activation to support mesenchymal TNBC cell migration and YAP is also required for focal adhesions activation.

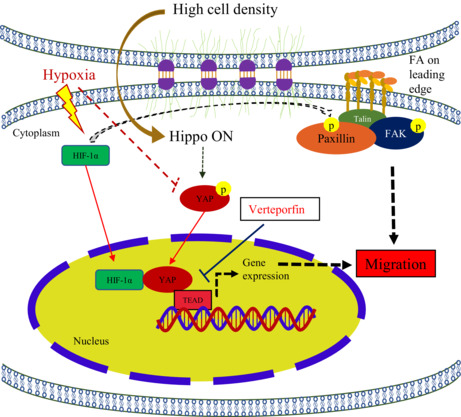

## 1 INTRODUCTION

Triple‐negative breast cancer (TNBC), an aggressive tumor that lacks estrogen receptor (ER), progesterone receptor (PR), and human epidermal growth factor receptor 2 (HER2),^1^ is the most common subtype causing mortality in breast cancer accounting for approximately 15%–20% of all breast cancers. Patients with TNBC have a high potential for distant metastasis, a higher rate of recurrence and worse prognosis than other subtypes.^2^ Although treatment strategies have been reported, TNBC remains the common cause of cancer‐related death, and treatment options remain a clinical challenge for scientists and oncologists.

Hypoxia is a fundamental process that occurs in the tumor microenvironment (TME) and plays a crucial role in tumor development and progression. At the subcellular level, hypoxia triggers a string of processes that promote tumor growth, control and regulate angiogenesis, enhance immune‐resistance responses, invasion, epithelial mesenchymal transition (EMT), induces tumor malignancy and metastasis.^3^ At the transcriptional level, the hypoxia response is primarily mediated by hypoxia‐inducible factors (HIFs), which are transcription factors. Hypoxia leads to HIF‐1α stabilization and HIF‐1α translocate to the nucleus and induce the expression of numerous genes.^4^, ^5^ Therefore, the abnormal stabilization of HIF‐1α affects the upregulation of its downstream targets. Overexpression of HIF‐1α has been positively correlated with poor prognosis and disease progression in many studies on patients with breast cancer.^6^, ^7^, ^8^, ^9^ Furthermore, activation of HIF‐1α induces several target genes of HIF‐1α that regulate multiple biological functions including proliferation, aggressive tumor phenotype, maintenance of the cancer stem cells (CSCs), and metastasis in TNBC.^10^, ^11^ HIF‐1α ubiquitination and degradation significantly reduced EMT and angiogenesis in TNBC cells.^12^

Yes‐associated protein (YAP) is a transcriptional factor that mainly regulates the downstream effector of the Hippo signaling pathway, which plays a key role in regulating various processes of cancer progression, particularly metastasis.^13^, ^14^ YAP can shuttle from the cytoplasm to the nucleus by inhibiting Hippo kinases.^15^ Hippo “OFF” leads to subsequent dephosphorylation of YAP, it translocates to the nucleus and binds to TEAD family transcription factors.^13^, ^15^ The YAP‐TEAD interaction induces expression of target genes such as *CTGF*, *CYR61*, *ITGB2*, *CCND1*, *AXL*, *DKK1*, *WWC1*, *AMOTL2*, *NPPB*, and *ANKRD1*.^16^, ^17^ YAP binds to TEAD in the nucleus and modulates cell growth, proliferation, metabolism, migration, invasion and metastasis.^18^, ^19^ Therefore, any alteration or mutation of YAP in cell is very important in cancer progression. Interestingly, increased YAP expression have been shown prevents DNA damage,^13^ enhances chemoresistance,^20^ and promotes cell migration and invasion in TNBC.^21^ An early study showed that SIAH2 promotes LATS ubiquitylation and degradation by E3 ubiquitin ligase, leadings to YAP nuclear translocation under hypoxia conditions.^22^ It has been reported that an increase in YAP activation by HIF‐2α under hypoxia conditions mediates cancer cell growth.^23^ GPRC5A directly transcripts of the target gene of HIFs lead to YAP activation under hypoxia.^24^ Hypoxic stress promotes YAP binding to HIF‐1α in the nucleus and sustains HIF‐1α protein stability to promote glycolysis in hepatocellular carcinoma cells.^25^ Nevertheless, the subcellular mechanism underlying YAP distribution under hypoxia conditions still remains poorly understood.

Paxillin is a central component of focal adhesions (FAs) that plays a critical role in the transduction of extracellular signals into intracellular responses. As a scaffolding protein, paxillin serves as a platform for the recruitment of specific kinases and phosphatases, cofactors, oncoproteins, and structural proteins, involved in conventional intracellular signaling pathways.^26^, ^27^ Paxillin plays a key role in recruiting FAs to the front of cells to support the assembly adhesion complex.^28^ Moreover, paxillin is required for FAs at the rear end of the cell facilitating trailing‐edge detachment during cell migration.^26^ Furthermore, upregulation of paxillin under hypoxia is dependent on HIF‐1α.^29^ Therefore, paxillin plays an important role in FA dynamics.

Tumor progression within a hypoxia tumor microenvironment causes cancer cells to undergo genetic and adaptive changes that allow them to survive. However, evidence of YAP activation in TNBC cells under hypoxia conditions is poorly understood. It is necessary to prove that high expression of YAP under hypoxia conditions may promote aggressive TNBC cells in facilitating the metastasis progression. In this study, we demonstrated that YAP is a novel effector for promoting mesenchymal TNBC cell migration under hypoxia conditions.

## 2 MATERIALS AND METHODS

### 2.1 Cell lines

MCF10A non‐malignant breast epithelial cells were cultured in high glucose Dulbecco's modified Eagle's medium (DMEM; GIBCO). MCF7 cells are human breast cancer cells that are positive for ER, PR and negative for HER2, and were cultured in high glucose DMEM. Five TNBC cell lines are negative for ER, PR and HER2 expression. MDA‐MB‐468 and HCC 1806 cells are basal‐like TNBC cells; MDA‐MB‐468 cells were cultured in high glucose DMEM, and HCC 1806 cells were cultured in RPMI‐1640 (GIBCO). BT‐549, MDA‐MB‐231, and Hs 578T cells are mesenchymal TNBC cells; BT‐549 cells were cultured in RPMI‐1640 containing 0.05 IU/mL of insulin transferrin selenium (ITS; GIBCO); MDA‐MB‐231, and Hs 578 T cells were cultured in high glucose DMEM. All cells were cultured in an incubator (5% CO_2_ at 37°C) with culture medium containing 10% fetal bovine serum (FBS; GIBCO), 100 IU/mL penicillin and streptomycin (P/S; GIBCO). For the hypoxia treatment, a hypoxia chamber and 400 μM CoCl_2_ were used. Hypoxia chambers were achieved with a proportion of O_2_/CO_2_ in incubator containing a gas mixture composed of 1% O_2_, 5% CO_2_ and 94% N_2_. Cobalt chloride (CoCl_2_) was commonly used as hypoxia‐mimicking agent. The cells were exposed to hypoxia conditions for 8 h and harvested for further analysis.

### 2.2 Reagents and antibodies

CoCl_2_ (#232696, Sigma‐Aldrich) and verteporfin (VP; #5305, Tocris Bioscience) were purchased from Sigma‐Aldrich and Bio‐Techne, respectively. The antibodies were used consist of HIF‐1α (#127309, GenTex), YAP (#H00010413‐M01, Abnova), phospho‐paxillin Tyr118 (#69363s, BD Biosciences), Paxillin (#611051, BD Biosciences), Hoechst 33342 (#H1399, Invitrogen), GAPDH (#100118, GenTex). The secondary antibody recognized the primary antibody consisting of AlexaFluor® 488 and AlexaFluor® 594 antibodies (#ab150077, #ab150113 and #ab150116, Abcam). The secondary antibody used for western blotting was horse radish peroxidase (HRP) conjugated IgG (#115‐035‐003 and 115‐035‐003, Jackson ImmunoResearch Laboratories).

### 2.3 Immunofluorescence staining

The cells were fixed with 4% cold paraformaldehyde (PFA) for 15 min, permeabilized with 0.5% TritonX‐100 in PBS for 10 min at room temperature and blocked with CAS‐Block™ Histochemical Reagent (#008120, Invitrogen) for 1 h at room temperature. The cells were then incubated overnight with the indicated antibodies at 4°C. After the antigen–antibody reaction, the cells were incubated with AlexaFluor® conjugated secondary antibodies, respectively, and the nuclear stain Hoechst 33342 for 1 h at 25°C. Immunofluorescence images were acquired using a FluorView laser scanning confocal microscope (FV3000, Olympus) equipped with 405, 488 and 543 nm lasers.

### 2.4 Cell proliferation assay

Cells were seeded and treated with 400 μM CoCl_2_ and 0.5 μM VP at different time points (0, 24, 48, 72 h). Cell proliferation was measured using the CCK8 (#3CK04‐11, Dojindo Molecular Technologies, Inc.) according to the manufacturer's instructions. Following incubation of the cells at 37°C in 5% CO_2_ for 2–4 h, the absorbance was measured at a wavelength of 450 nm using a FlexStation 3 Microplate Reader (Molecular Devices).

### 2.5 Time‐lapse recording of living cells

Cells were seeded into 3 cm dishes for 3 h, followed by treatment with 400 μM CoCl_2_ and 0.5 μM VP and maintained at 37°C in 5% CO_2_ throughout the recording time. The mini‐image system (Lumascopye 520, Etaluna) was applied to capture the images at 1‐min intervals for 4 h. Finally, automated cell tracking and analysis in phase‐contrast videos were performed using the iTrack4U software.

### 2.6 Transwell migration assay

Cells were seeded into 8 μm‐pore transwell inserts in serum free medium. The lower wells were filled with medium supplemented with 3% FBS as a chemoattractant and then incubated for 8 or 12 h with 400 μM CoCl_2_ and 0.5 μM VP. The cells were fixed with 100% methanol for 2 min at 25°C and stained with 0.1% crystal violet for 30 min. Transwell cells were obtained by using bright‐field microscopy and counted using ImageJ software.

### 2.7 Wound healing assay

Cells were seeded with an equal number of cells in two wells of culture‐inserts (#81176, ibidi) and incubated overnight at 37°C in 5% CO_2_ The inserts were removed and treated with 400 μM CoCl_2_, and the wound area was taken at 0, 6, 12, 24 h using a phase‐contrast microscope. The wound area was measured using ImageJ software.

### 2.8 Cell polarization assay

Cells were counted and seeded into 3 cm dishes in culture medium at 37°C in 5% CO_2_ for different time points (4, 6, 12 h). Cell morphology was taken using bright‐field microscopy.

### 2.9 Focal adhesion dynamics under TIRF microscopy

Cells were transfected with the GFP‐paxillin plasmid using Lipofectamine™ 3000 Transfection Reagent (#L3000015, Thermo Fisher Scientific) according to the manufacturer's protocol. A total of 24–48 h after transfection, the dynamics of paxillin under normoxia and hypoxia conditions were immediately recorded. Paxillin was clearly observed in the 100 nm evanescent wave of the 488 nm laser. Images were taken every 30 s for 1 h using a total internal reflection fluorescence (TIRF) microscope (cellTIRF, Olympus).

### 2.10 Protein quantification and immunoblotting

The extract lysate comprising an equal amount of protein lysates were prepared and separated by SDS‐PAGE gel and electroblotted onto a nitrocellulose membrane. Membranes were blocked with 5% non‐fat milk, followed by incubation with the indicated antibodies. Immune complexes were detected using horseradish peroxidase‐conjugated IgG. Finally, ECL was used to visualize the targeted proteins using an ImageQuant™ LAS 4000 system (GE Healthcare).

### 2.11 Statistical analysis

All experiments were independently performed at least three times. SigmaStat Statistical Software 17.0 (SPSS Inc.) was used for all the statistical analyses. The results are shown as mean ± SEM (standard error mean). Differences between the two groups were assessed using an unpaired, two‐tailed Student's t‐test. The Bonferroni post‐hoc t‐test was performed to make statistical comparisons in multi‐group analysis after a significant result was obtained using ANOVA. *p < 0.05, **p < 0.01, ***p < 0.001.

## 3 RESULTS

### 3.1 Hypoxia‐induced YAP nuclear translocation at high cell density in normal, non‐TNBC and mesenchymal TNBC cells

**FIGURE 1:**
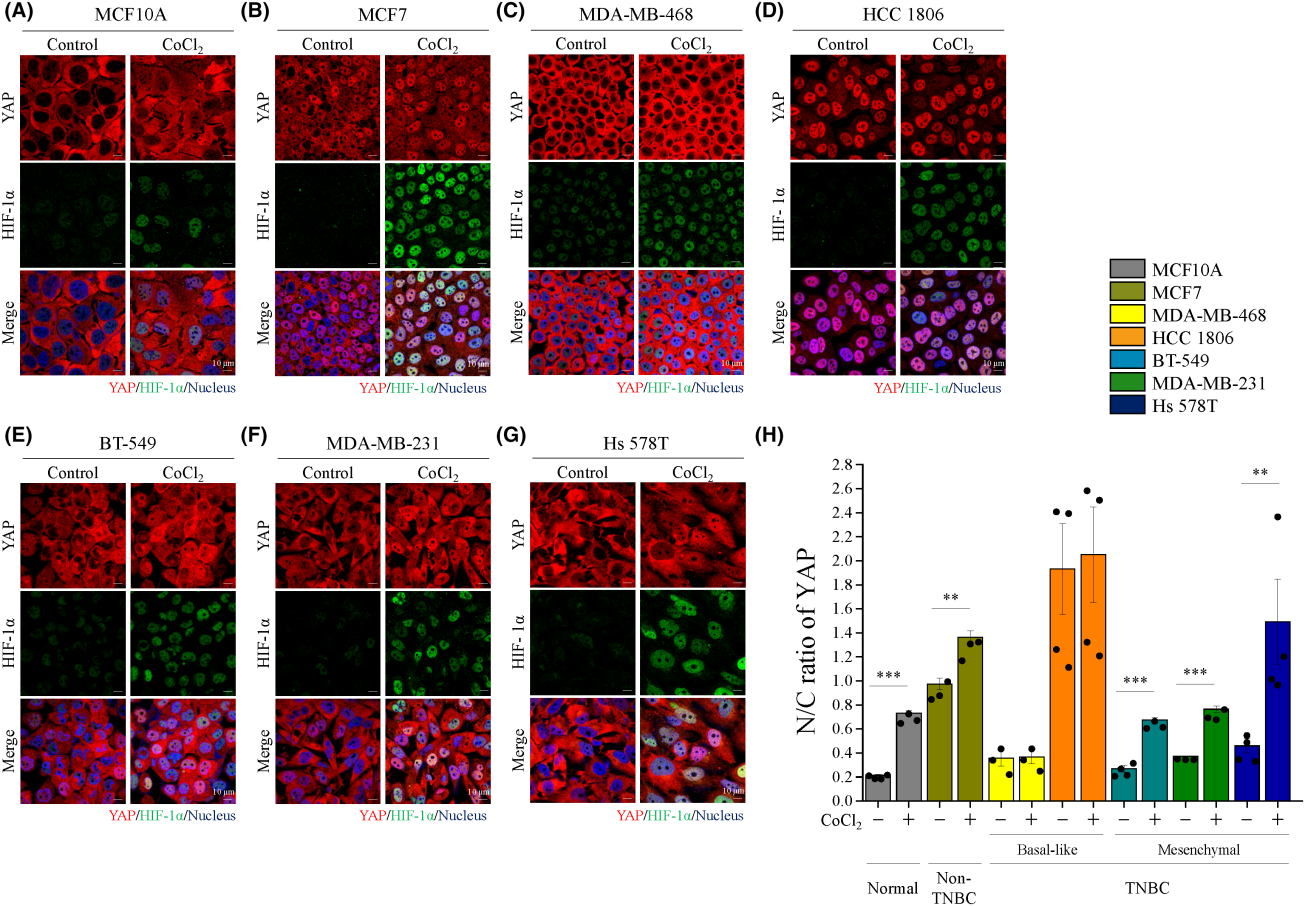
Hypoxia‐induced YAP nuclear translocation at high cell densities in variety of breast cell lines. Seven cell lines were seeded at high cell density and treated with or without 400 μM CoCl_2_. Immunofluorescence staining of YAP (red), HIF‐1α (green) and nuclear Hoechst 33342 (blue) by confocal microscopy. (A–G) Representative images of seven cell lines (A) MCF10A, (B) MCF7, (C) MDA‐MB‐468, (D) HCC 1806, (E) BT‐549 (F) MDA‐MB‐231, and (G) Hs 578T. Scale bars, 10 μm. (H) Quantitative analysis of the N/C ratio of YAP. Data are expressed as mean ± SEM values of at least three independent experiments. *p < 0.05, **p < 0.01, ***p < 0.001. N: intensity in nuclei, C: intensity in cytoplasm.

First, we assessed the influence of hypoxia on the expression of YAP in seven breast cell lines. We stimulated the hypoxia condition of these cells under starvation‐serum at high cell density using a hypoxia chamber and the hypoxia‐mimicking agent CoCl_2_. We observed that cytoplasmic accumulation of YAP was clearly shown in MCF10A, MDA‐MB‐468, BT‐549, MDA‐MB‐231, and Hs 578T cells (Figure 1A,C,E–G) and a part of YAP in nucleus in MCF7 cells (Figure 1B) under normoxia conditions. Interestingly, YAP was significantly translocated into the nucleus in MCF10A, MCF7, BT‐549, MDA‐MB‐231, and Hs 578T cells (Figure 1A,B,E–G) under CoCl_2_ treatment. However, CoCl_2_ had no effect on the basal‐like TNBC type consisting of MDA‐MB‐468 and HCC 1806 cells (Figure 1C,D). Indeed, the ratio of nucleus/cytoplasmic YAP significantly increased with CoCl_2_ treatment (Figure 1H). Furthermore, hypoxia chamber also showed similar results (Figure S1). Immunoblotting showed that hypoxia significantly increased HIF‐1α protein expression level in the seven breast cancer cell lines (Figure S2). These data are consistent with the immunofluorescence images showing that HIF‐1α is highly expressed under hypoxia conditions (Figure 1A–G and Figure S1). Moreover, the expression of YAP in nuclear extracts similarly showed the same phenomenon of increased YAP nuclear translocation by hypoxia stimulation in Hs 578T cells (Figure S3). These data strongly demonstrate that hypoxia‐induced YAP nuclear translocation occurred at high cell density in normal cells, non‐TNBC cells, and mesenchymal cells, but had no effect in basal‐like TNBC cells.

### 3.2 YAP activation may not associate for cell proliferation during hypoxia stimulation

**FIGURE 2:**
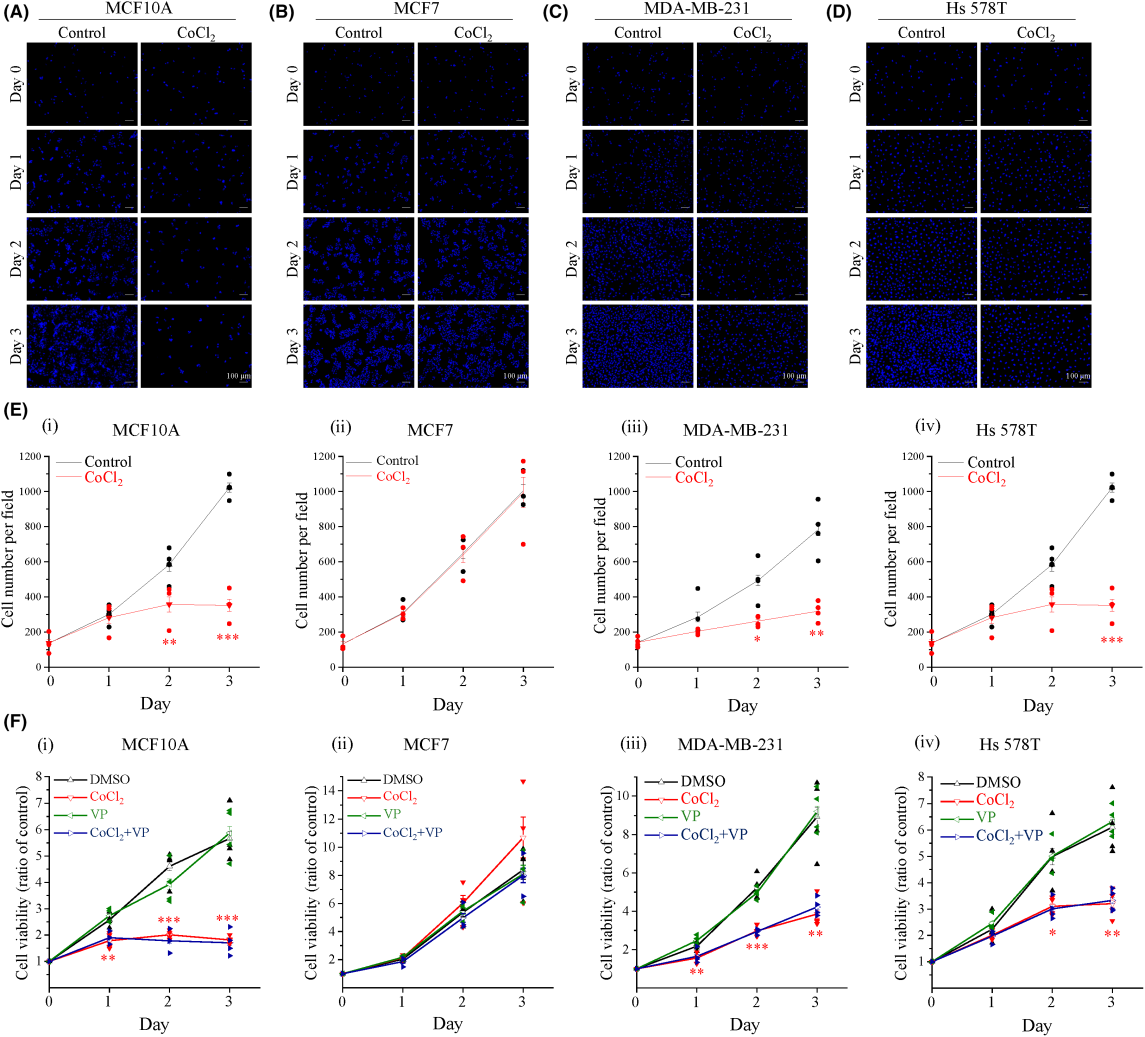
Hypoxia may participate in inhibition of cell proliferation but may not correlate with YAP activation. Cells were treated with or without 400 μM CoCl_2_. (A‐D) Nuclei showed in blue. Scale bars, 100 μm. Representative images of (A) MCF10A, (B) MCF7, (C) MDA‐MB‐231, and (D) Hs 578T cells. (E) Quantification of the cell number per field. CCK8 assays were performed with or without 400 μM CoCl_2_ and 0.5 μM VP in four cell lines. (F) Cell viability of the time indicated. Data points represent mean ± SEM (*n* ≥ 3). *p < 0.05, **p < 0.01, ***p < 0.001. VP, verteporfin.

Next, we proposed that YAP activation might correlate with cell growth. The fluorescence images and analysis data showed that CoCl_2_ treatment significantly inhibited the cell growth of MCF10A, MDA‐MB‐231, and Hs 578T cells (Figure 2A,C–E). However, the CoCl_2_ treatment had no effect on MCF7 cells (Figure 2B,E). Cell proliferation assay was performed to further investigate the relationship between YAP activation and cell proliferation after CoCl_2_ treatment. Similarly, cell viability was significantly reduced in MCF10A, MDA‐MB‐231, and Hs 578T cells, but did not significantly affect MCF7 cells (Figure 2F). To evaluate whether YAP nuclear translocation reduces cell growth during hypoxia, we treated the cell with VP, a YAP inhibitor that has been shown to inhibit the YAP‐TEAD interaction.^16^, ^30^ As results shown in Figure 2F, VP did not significantly affect cell growth under either normoxia or hypoxia in any of the four cell lines. These data indicate that YAP may not be correlated with reduced cell proliferation caused by hypoxia.

### 3.3 YAP nuclear accumulation is positively correlated with hypoxia‐promoting mesenchymal TNBC cell migration

**FIGURE 3:**
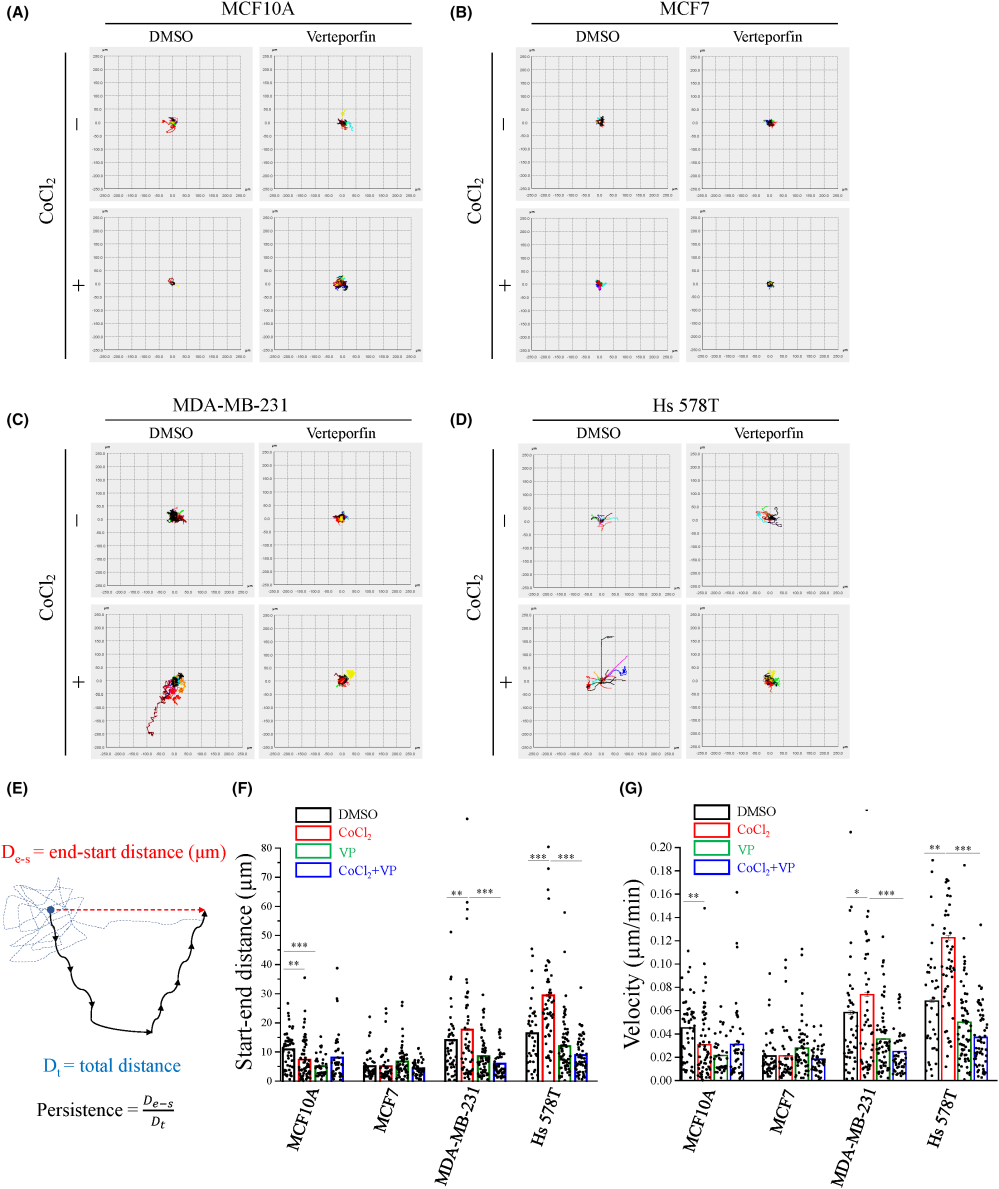
Hypoxia promotes YAP nuclear accumulation to facilitate mesenchymal TNBC cell migration. Time‐lapse recording of living cell was performed to evaluate 2D single‐cell migratory ability under with or without 400 μM CoCl_2_ and 0.5 μM VP. (A–D) Overall migration trajectories of individual cells of one representative experiment of (A) MCF10A, (B) MCF7, (C) MDA‐MB‐231, and (D) Hs 578T cells. Each color represents a single cell. X and Y axis are expressed distance (μm). (E) The schematic illustrates the definition of total distance (blue dash line), start‐end distance (red dash line) and persistence. (F, G) Quantitative analysis of (F) start‐end distance (μm) and (G) velocity (μm/min). Data were collected from three independent experiments and at least 40 cells. Data are expressed as mean ± SEM (*n* ≥ 3). *p < 0.05, **p < 0.01, ***p < 0.001. TNBC, triple‐negative breast cancer; VP, verteporfin.

**FIGURE 4:**
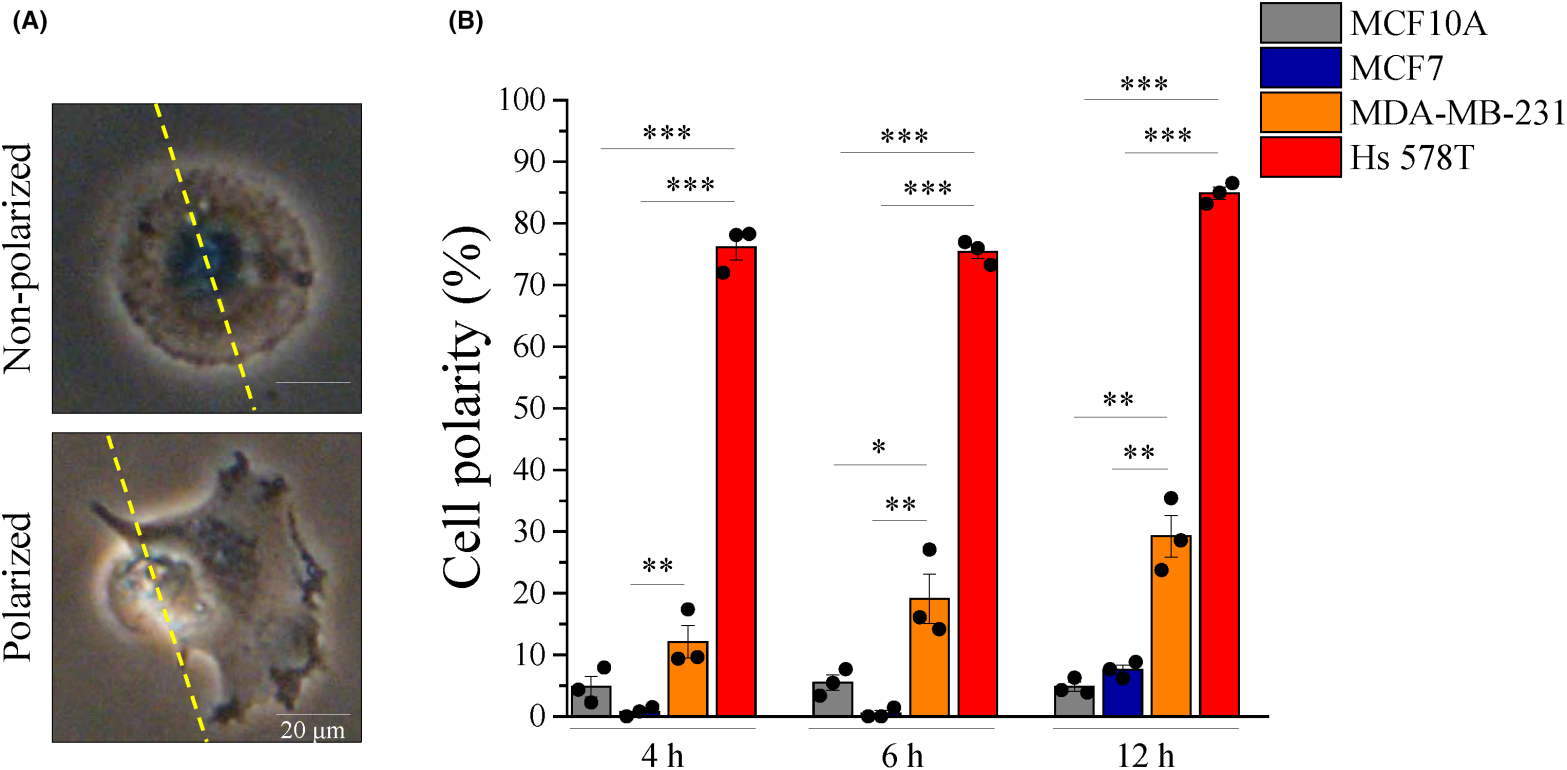
Mesenchymal TNBC cells express higher cell polarized ability. (A) Representative images of non‐polarized cells (upper panel) and polarized cells (lower panel). Scale bars, 50 μm. (B) Quantitative analysis of the percentage of polarity cell at independent times. Data are expressed as mean ± SEM values of three independent experiments. *p < 0.05, **p < 0.01, ***p < 0.001. TNBC, triple‐negative breast cancer.

To explore the potential function of hypoxia‐induced YAP activation, we monitored the track of four cell lines during hypoxia stimulation. The images shown that the trajectories in MDA‐MB‐231 and Hs 578T cells were greater and straighter than those in MCF10A and MCF7 cells under hypoxia (Figure 3A–D). However, the trajectories of MCF10A cells were shorter under hypoxia condition (Figure 3A). Cell migration requires cell polarization and the formation of protrusions at one end of the cell, leading to the question of the relationship between cell polarity and directional migration. The yellow dashed line that crosses the middle of the nucleus of the cells is used as a reference line. Our definition of a polarized cell was based on the display of an extended lamellipodium on one side of the reference line. In contrast, a non‐polarized cell was defined with no difference in lamellipodium contributing to protrusion on the two sides of the reference (Figure 4A). The analysis of polarized cells showed that MDA‐MB‐231 and Hs 578T cells were highly polarized compared to MCF10A and MCF7 cells at different time points after seeding (Figure 4B). These data might indicate that mesenchymal TNBC cells have a greater migration potential, and the direction of their migration is straightforward from the original position under hypoxia conditions. Next, we evaluated the influence of YAP on these properties of cells under hypoxia conditions. Under hypoxic conditions, the migratory ability of MDA‐MB‐231 and Hs 578T cells was significantly increased. However, MDA‐MB‐231 and Hs 578T cells in hypoxia were profoundly sensitive to VP compared with normoxia conditions (Figure 3C,D). The trajectories migration in MDA‐MB‐231 and Hs 578T cells with the VP treatment was clearly reduced during the hypoxia (Figure 3C,D). Start‐end distance was defined as the shortest distance between the initial and final positions of the cell (Figure 3E). Indeed, the analysis of start‐end distance showed that hypoxia significantly increased, and VP treatment remarkably decreased the start‐end distance cell migration in MDA‐MB‐231 and Hs 578T cells. However, not impacted to MCF7 cells (Figure 3F). Furthermore, the velocity of these two mesenchymal TNBC cells were accelerated under hypoxia conditions, and VP significantly reduced the effect of hypoxia, whereas hypoxia remarkably decreased the velocity of MCF10A cells (Figure 3G). Collectively, these data indicate that YAP activation promotes migration of mesenchymal TNBC cells under hypoxia conditions.

### 3.4 YAP activation is required for hypoxia‐promoting mesenchymal TNBC cell migration

**FIGURE 5:**
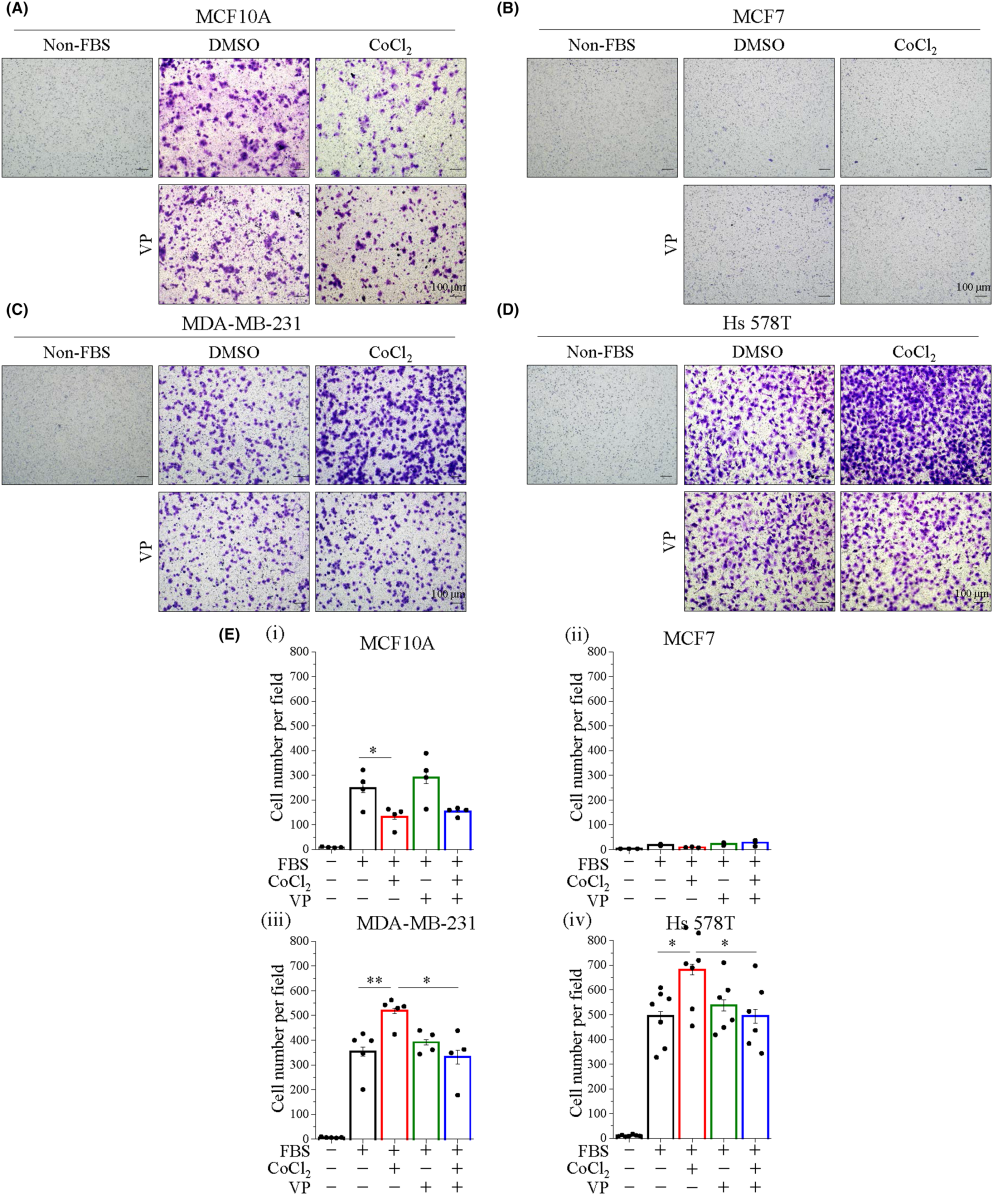
YAP is required for hypoxia‐stimulated mesenchymal TNBC cell migration. Transwell assay was performed to evaluate 3D single‐cell migratory ability under with or without 400 μM CoCl_2_ and 0.5 μM VP. (A–D) Representative microscopic images of cells migrated of (A) MCF10A, (B) MCF7, (C) MDA‐MB‐231, and (D) Hs 578T cells. The medium without FBS was used as a negative control. Scale bars, 100 μm. (E) Quantitative analysis of the number of cells transwell per field. Data are expressed as mean ± SEM values of at least three independent experiments. *p < 0.05, **p < 0.01, ***p < 0.001. FBS, fetal bovine serum; VP, verteporfin.

To validate the relationship between YAP activation and migration in mesenchymal TNBC cells, we further performed a transwell assay to investigate the 3D‐migration ability through a microporous membrane. Fetal bovine serum (FBS) increased the number of transwell cells in MCF10A, MDA‐MB‐231, and Hs 578T cells, but not in MCF7 cells (Figure 5A–D). Interestingly, hypoxia increased cell migration in MDA‐MB‐231 and Hs 578T cells, respectively (Figure 5C–E), whereas it decreased MCF10A cell migration (Figure 5A,E). These data reveal that hypoxia is beneficial for TNBC cell migration. Moreover, VP treatment remarkably decreased the effect of hypoxia, which induced MDA‐MB‐231 and Hs 578T cell migration (Figure 5C–E). Notably, MCF7 cells without cell migration in the 2D and 3D migration assays (Figures 3B and 5B,E). Furthermore, we treated cells in the hypoxia chamber and obtained the same results (Figure S4). Taken together, these data indicate that YAP is required for migration of mesenchymal TNBC cells under hypoxia conditions.

### 3.5 Collective cell migration is not mediated by hypoxia‐induced YAP nuclear translocation

**FIGURE 6:**
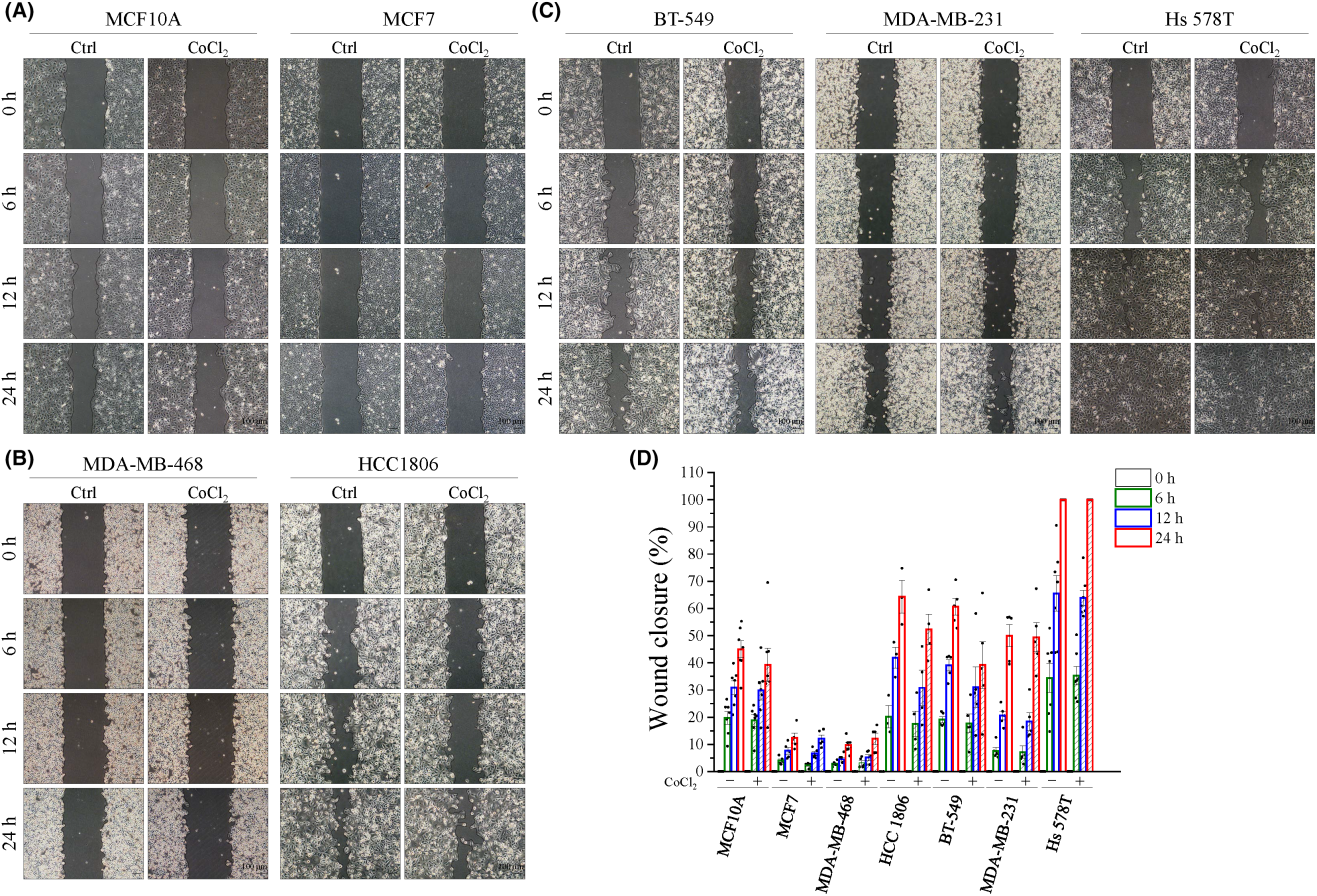
Hypoxia would not promote collective cell migration. Wound healing assay was performed to evaluate collective cell migratory ability with or without 400 μM CoCl_2_. (A–C) Representative images of (A) MCF10A and MCF7 cells, (B) basal‐like TNBC: MDA‐MB‐468 and HCC 1806 cells, (C) mesenchymal TNBC: BT‐549, MDA‐MB‐231, and Hs 578T cells. Scale bars, 100 μm. (D) Quantitative analysis of migratory ability is presented as the percentage of wound closure. Data are expressed as mean ± SEM values of at least three independent experiments.

Cancer cells usually use different migration strategies, consisting of individual and collective movements.^31^ These two strategies can be converted like bidirectional transitions. First, we observed that while MCF10A, MCF7 and basal‐like TNBC cells consisted of MDA‐MB‐468 and HCC1806 cells exhibiting a group cell pattern, mesenchymal TNBC cells displayed a mesenchymal or spindle shape (Figure S5). A group cell migration assay was performed to verify whether hypoxia‐induced YAP activation affected collective cell migration. We observed that mesenchymal TNBC cells closed the wound faster than MCF10A, MCF7, and MDA‐MB‐468 cells in normal conditions (Figure 6A–D), whereas HCC 1806 cells still showed a wound area similar to that of BT‐549 and MDA‐MB‐231 cells, probably because HCC 1806 cells showed higher expression of nuclear YAP even under normoxia conditions (Figures 1D and 6C,D). However, hypoxia did not affect wound closure at the same time points as normoxia (Figure 6D). Collectively, these data indicate that hypoxia did not affect collective group cell migration.

### 3.6 YAP activation is positively associated with hypoxia promotes FAs activation

**FIGURE 7:**
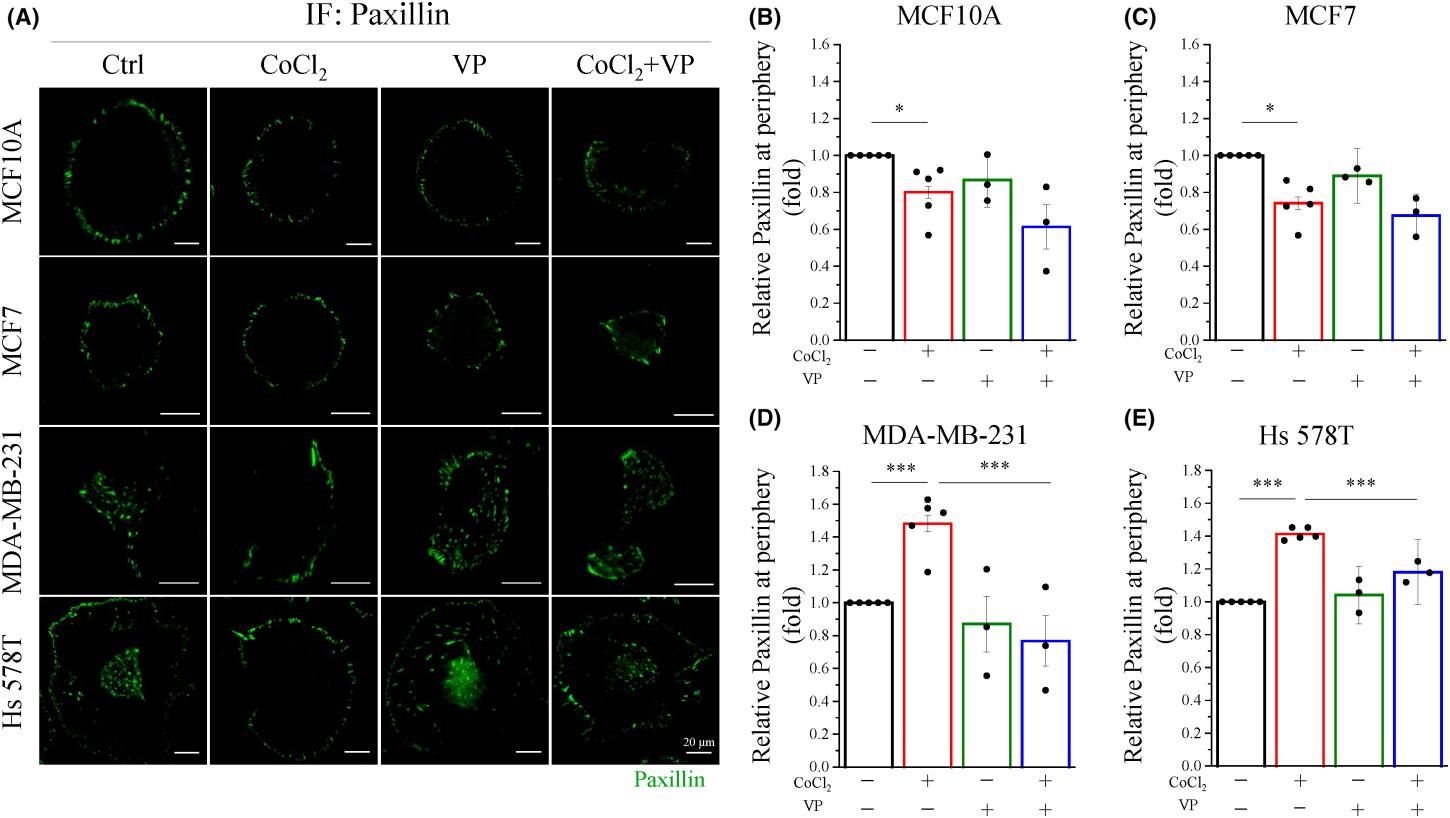
Hypoxia‐induced focal adhesion expression in the leading edge is mediated by YAP activation. Cells were seeded and treated with or without 400 μM CoCl_2_ and 0.5 μM VP (A) Representative image of Paxillin (green) by TIRF microscopy. Scale bars, 20 μm. (B–E) Quantitative analysis related of paxillin on the leading edge of (B) MCF10A, (C) MCF7, (D) MDA‐MB‐231, and (E) Hs 578T cells. Data are expressed as mean ± SEM values of at least three independent experiments. *p < 0.05, **p < 0.01, ***p < 0.001. TIRF, total internal reflection fluorescence.

**FIGURE 8:**
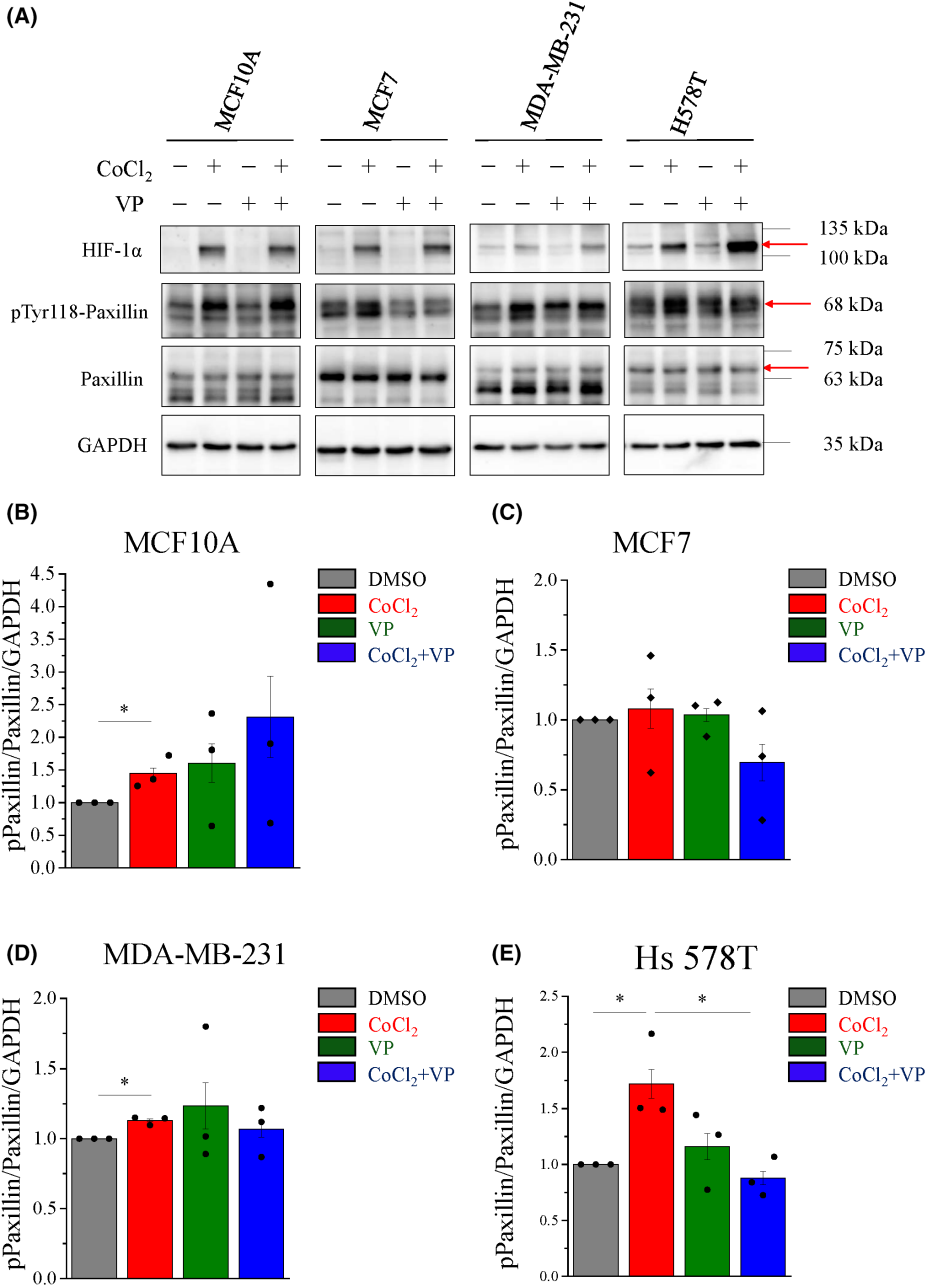
YAP plays critical role for hypoxia‐activated focal adhesion in mesenchymal TNBC cells. Cells were seeded and treated with or without 400 μM CoCl_2_ and 0.5 μM VP (A) Representative immunoblots of the phosphorylated paxillin (pTyr118‐Paxillin), Paxillin and HIF‐1α were shown, and GAPDH was used as an internal control in cell lines. (B–E) Quantitative analysis of the Paxillin/Paxillin ratio normalized to GAPDH of (B) MCF10A, (C) MCF7, (D) MDA‐MB‐231, and (E) Hs 578T cells. Data are expressed as mean ± SEM values of three independent experiments. *p < 0.05. TNBC, triple‐negative breast cancer; VP, verteporfin.

FA assembly, turnover, and dynamics play critical roles in cell migration, which requires coordinate formation at the cell periphery, adhesion disassembly and retraction at the rear trailing edge.^32^ Thus, we hypothesized that hypoxia promotes mesenchymal TNBC migration, which might be associated with FAs activation. As a major FAs participating in modulating cell mobility, paxillin recruits nascent FAs at the cell periphery for the assembly of adhesion complexes.^26^ GFP‐Paxillin was transfected into two mesenchymal TNBC cells and then monitored for the dynamics of paxillin during hypoxia stimulation under TIRF microscopy. At 0‐min, paxillin appeared in the whole cell, and it quickly alternated the localization to the cell periphery after 1 h of hypoxia treatment in both MDA‐MB‐231 and Hs 578T cells (Figure S6). In addition, we performed immunofluorescence staining under TIRF microscopy to evaluate the distribution and proportion of paxillin after hypoxia stimulation. Similar to exogenous GFP‐paxillin, immunofluorescence staining of endogenous paxillin was observed in all MDA‐MB‐231 and Hs 578T cells in normoxia conditions, it was only located at the cell periphery membrane under hypoxia conditions. However, the distribution of paxillin did not change in MCF10A and MCF7 cells under hypoxia stimulation (Figure 7A). Indeed, the analysis of the paxillin intensity at the periphery membrane per cell remarkably increased in MDA‐MB‐231 and Hs 578T cells, but significantly decreased in MCF10A and MCF7 cells under hypoxia conditions (Figure 7B–E). These data indicated that hypoxia triggers focal adhesion turnover in mesenchymal TNBC cells. Interestingly, VP significantly inhibited paxillin only expression at the cell periphery membrane in both MDA‐MB‐231 and Hs 578T cells, whereas there was no difference in both MCF10A and MCF7 cells under hypoxia stimulation (Figure 7B–E). In addition, the function and localization of FA are strongly regulated by paxillin phosphorylation, we performed immunoblotting to further test our hypothesis. Hypoxia‐induced phosphorylation of paxillin at Tyr118 in MCF10A, MDA‐MB‐231, and Hs 578T cells, but it had no effect on MCF7 cells (Figure 8A–E). In particularly, VP significantly inhibited the phosphorylation of paxillin at Tyr118 in Hs 578T cells under hypoxia conditions (Figure 5A,E). Taken together, these data indicate that YAP is positively correlated with FAs activation under hypoxia conditions.

## 4 DISCUSSION

Unlike to other conventional signaling pathways, YAP signaling is unique because it does not affect the ligand bond or receptor system. Instead, YAP is affected by numerous factors in the TME such as cell–cell junction, cell morphology, cell polarity, cell‐ECM interactions, and mechanical cues.^19^ Many studies have shown that increased cell density induces YAP cytoplasmic retention.^33^ We have also shown YAP cytoplasmic localization at high cell density in normoxia conditions. Interestingly, hypoxia‐induced HIF‐1α expression simultaneously triggered YAP nuclear translocation in non‐malignant breast cells, non‐TNBC cells and mesenchymal TNBC cells. Whereas hypoxia had no impact on basal‐like TNBC cells (Figure 1 and Figure S1). Accordingly, hypoxia‐induced YAP nuclear translocation and activation has also been reported that contributes to promote the resistance of SN38 in hepatocellular carcinoma cells.^34^ Consistent with the results of previous studies, we also observed YAP nuclear accumulation under hypoxia. However, we discuss the effect of hypoxia on YAP expression in TNBC cells.

In mammals, facilitating YAP stability induces its target gene expression, which promotes the migration, invasion, and progression of TNBC cells.^21^, ^35^ Functional investigation revealed that hypoxia significantly increased the net distance of individual cell migration in mesenchymal TNBC cells (Figure 3C,D,F). In particularly, our data showed that YAP activation was required to increase the net distance of individual cell migration under hypoxia stimulation (Figure 3F). Metastasis is a process includes many steps, starting with the migration of the individual cancer cell from the primary tumor. In addition, individual cell migration has been shown to be related to distant metastasis.^36^, ^37^, ^38^ Intriguingly, hypoxia significantly increased the number of migratory cells in the transwell assay, which was only observed in mesenchymal TNBC cells. YAP played a critical role in the migration of mesenchymal TNBC cells under hypoxia conditions (Figure 5C–E and S4C‐E). Noticeably, we observed quite an interesting phenomenon that Hs 578T cells showed higher YAP nuclear translocation and it also showed high net distance of cell migration and high migratory ability under hypoxia conditions (Figures 1G,H, 3F and 5D,E). These data strongly suggested that YAP activation is required for the migration of mesenchymal TNBC cells under hypoxia conditions.

To migrate, cells first acquire a characteristic polarized morphology in response to extracellular signals.^39^ Observation of the cells showed that TNBC cells exhibited a highly polarized morphology compared to other cells (Figure 4B). In addition, cell migration commonly displays two modes consist of individual and collective movement. We found that hypoxia did not significantly affect collective cell migration, even in mesenchymal TNBC cells (Figure 6). As a migration strategy, mesenchymal cell migration is featured by individual cell motility.^38^

Formation of FAs at the leading edge plays a role as traction against the generation of tensional forces to push cell migration forward.^26^, ^40^ We found that hypoxia significantly induced the localization of paxillin at the leading edge in mesenchymal TNBC cells, whereas paxillin was present the entire cell in normoxia conditions. In contrast, the YAP inhibitor remarkably reduced the distribution of paxillin at the leading edge of mesenchymal TNBC cells under hypoxia conditions (Figure 7D,E). In specially, our results demonstrated that VP significantly inhibited hypoxia‐induced paxillin activation in Hs 578T cells (Figure 8A,E). In the FAs formation stage, paxillin phosphorylate at Tyr 118 plays a role in FAs dynamics during cell migration.^41^, ^42^ At the leading edge, Tyr31/118 phosphorylated paxillin induced FAs turnover simultaneously activates Rac1 and suppresses RhoA activity during cell migration.^43^ Moreover, hypoxia increased Rab5 activation to re‐localize FAs at the leading edge driven cell migration by FAK phosphorylation and Rac1 activation in MDA‐MB‐231 cells.^44^ Shen et al. demonstrated that thrombospondin 1 (THBS1) is a downstream of FA, which is a direct transcriptional target gene of YAP‐TEAD.^45^ Our data suggested that YAP is associated with hypoxia and promotes FAs activity in mesenchymal TNBC cells.

Many studies have reported that overexpression of the YAP oncoprotein promotes proliferation and survival in various cancer cell lines, especially in breast cancer.^46^ Moreover, hypoxia has been shown significantly promotes breast cancer cell proliferation.^47^ However, our results showed that hypoxia did not increase cell growth. In contrast, hypoxia significantly inhibited the growth on mesenchymal TNBC cells after 2 days of treatment (Figure 2). The effects of continuous hypoxia have been shown to inhibit the proliferation ability of MDA‐MB‐231 cells over 2 days, and intermittent hypoxia also significantly reduced cell proliferation in MDA‐MB‐231 cells.^48^ Furthermore, YAP activation had no effect on cell proliferation under hypoxia conditions following VP treatment (Figure 2F). These results may indicate that hypoxia promotes YAP activation is not associated with TNBC cell proliferation.

Our future work will explore whether hypoxia‐mediated HIF‐1α promotes nuclear YAP acts in mesenchymal TNBC cell lines and whether the mechanism HIF‐1α and YAP are transcriptional co‐activators under hypoxia conditions that regulate many steps of TNBC metastasis such as invasion and extracellular vesicle release during the cell migration and invasion. Currently, many YAP inhibitor compounds have been reported to inhibit YAP excluding VP such as TED‐347 and CA3.^18^, ^49^, ^50^, ^51^ In the future studies, we will further investigate the effect of other YAP inhibitors as well as siRNA against YAP on hypoxia‐induced mesenchymal TNBC migration.

In this study, we provide evidence that YAP as a key mediator of hypoxia‐induced mesenchymal TNBC cell migration based on the following evidence: (1) hypoxia triggers YAP nuclear translocation in mesenchymal TNBC; (2) hypoxia only promotes mesenchymal TNBC cell migration, and YAP plays a crucial role in cell migration under hypoxia; and (3) hypoxia promotes FAs activation to support mesenchymal TNBC cell migration. Interestingly, YAP is also required for FAs activation in Hs 578T cells, which are the most aggressive TNBC cell line. These data not only further provide evidence that hypoxia promotes TNBC cell migration, but also create exciting opportunities and challenges for understanding the progression of TNBC cells.

## AUTHOR CONTRIBUTIONS

**Thi My Hang Nguyen:** Conceptualization (lead); data curation (equal); investigation (lead); validation (equal); writing – original draft (lead). **Yi‐Shyun Lai:** Investigation (equal); methodology (supporting); validation (supporting). **Ying‐Chi Chen:** Investigation (supporting); methodology (supporting); software (supporting); validation (supporting). **Tzu‐Chien Lin:** Methodology (supporting); software (supporting). **Ngoc Thang Nguyen:** Investigation (supporting); methodology (supporting). **Wen‐Tai Chiu:** Conceptualization (equal); data curation (supporting); funding acquisition (lead); project administration (lead); resources (lead); supervision (lead); writing – original draft (supporting); writing – review and editing (lead).

## FUNDING INFORMATION

This work was supported by the National Science and Technology Council of Taiwan [110‐2628‐B‐006‐030 and 111‐2320‐B‐006‐058].

## CONFLICT OF INTEREST STATEMENT

The authors declare no competing interests.

## ETHICS STATEMENT

Approval of the research protocol by an Institutional Reviewer Board: Not applicable. Informed Consent: Not applicable. Registry and the Registration No. of the study/trial: Not applicable. Animal Studies: Not applicable.

## Supporting information


Figure S1‐S6

## Data Availability

Data sharing is not applicable to this article as no new data were created or analyzed in this study.
