# Supplementary material for: Hypoxia‐induced YAP activation and focal adhesion turnover to promote cell migration in mesenchymal TNBC cells
Source: Cancer Med. 2023 Feb 9;12(8):9723–37. doi: 10.1002/cam4.5680 (PMC10166962; doi:10.1002/cam4.5680)
Supplement: Supplementary file 1 — Figure S1‐S6 [file CAM4-12-9723-s001.pdf]

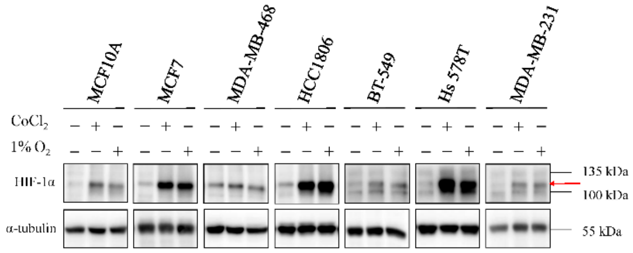

FIGURE S1 Hypoxia-mediated activation of HIF-1α. Immunoblotting assays were performed to identify that hypoxia upregulates HIF-1α expression. The cells were seeded at high cell density overnight, following cell attachment and growth in medium with 3% FBS, they were treated with or without 400 µM $CoCl_2$ and hypoxia chamber with 1% O_2. Cell lysates were immunoblotted to detect HIF-1α protein levels. Representative immunoblots of the HIF-1α. α-tubulin was used as an internal control. FBS, fetal bovine serum.

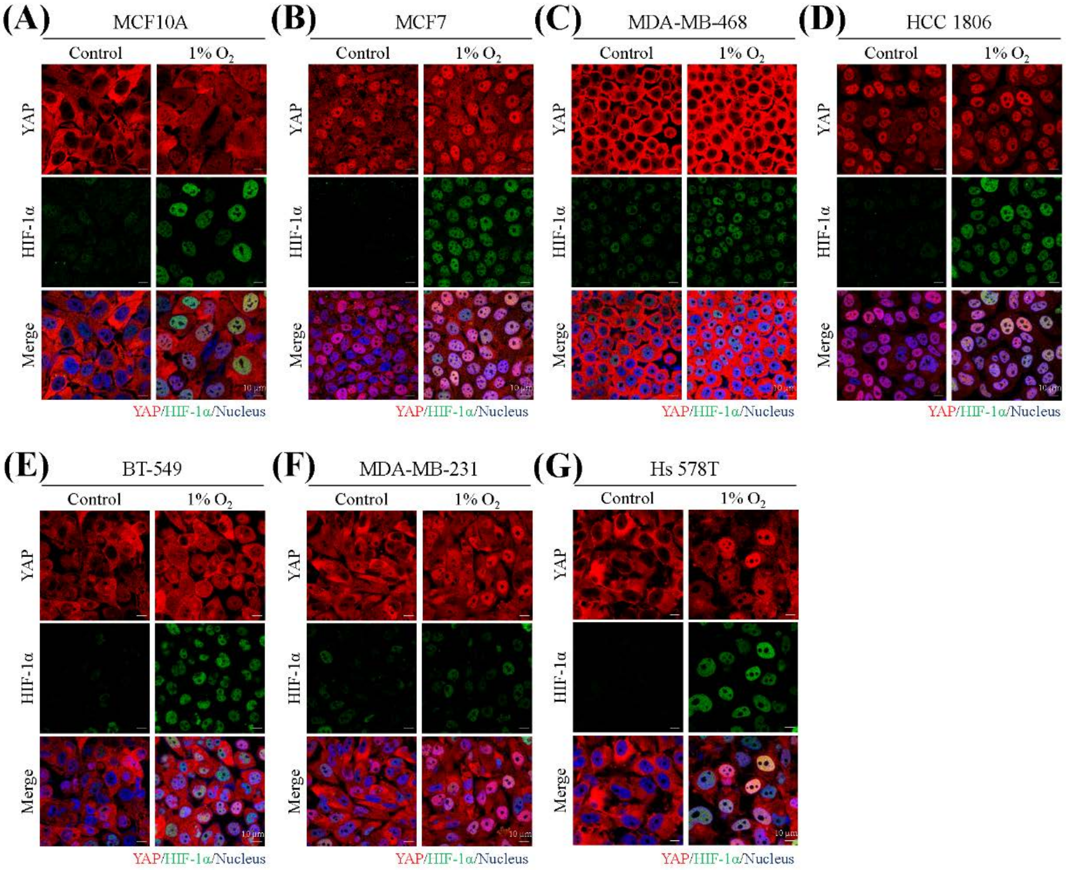

FIGURE S2 Hypoxia-induced YAP nuclear accumulation at high cell density in a variety of breast cell lines. Immunofluorescence assays were performed to further confirm the effect of hypoxia on YAP expression by using hypoxia chamber with 1% O_2. Seven cell lines were seeded at high cell density and incubated in normoxia or hypoxia chamber for 8 h. Immunofluorescence confocal images AlexaFluor 594 (red) for YAP, Alexa Fluor 488 (green) for HIF-1α, Hoechst 33342 was used to stain nucleus. Representative images of (A) MCF10A, (B) MCF7, (C) MDA-MB-468, (D) HCC 1806, (E) BT-549, (F) MDA-MB-231, and (G) Hs 578T cells. Scale bars, 10 µm. Data were collected from at least three independent experiments.

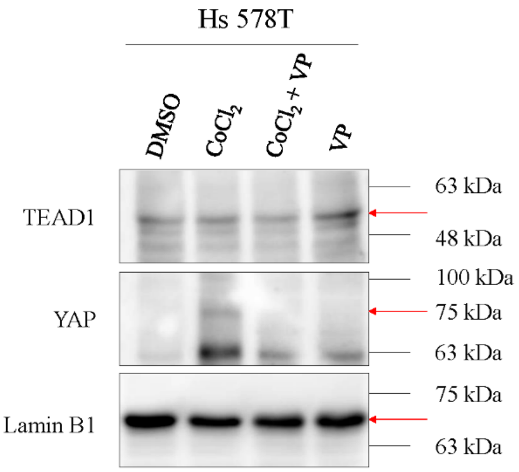

FIGURE S3 Hypoxia-induced YAP nuclear translocation. Immunoblotting was performed to further demonstrate that hypoxia promotes YAP nuclear translocation. Hs 578T cells were seeded at high cell density and treated with or without 400 µM $CoCl_2$ and 0.5 µM VP for 8 h. The nucleus extraction was collected and immunoblotted to detect YAP and TEAD-1 protein levels. Representative immunoblots of the YAP and TEAD-1 are shown. Lamin B1 was used as a marker of nuclear extracts.

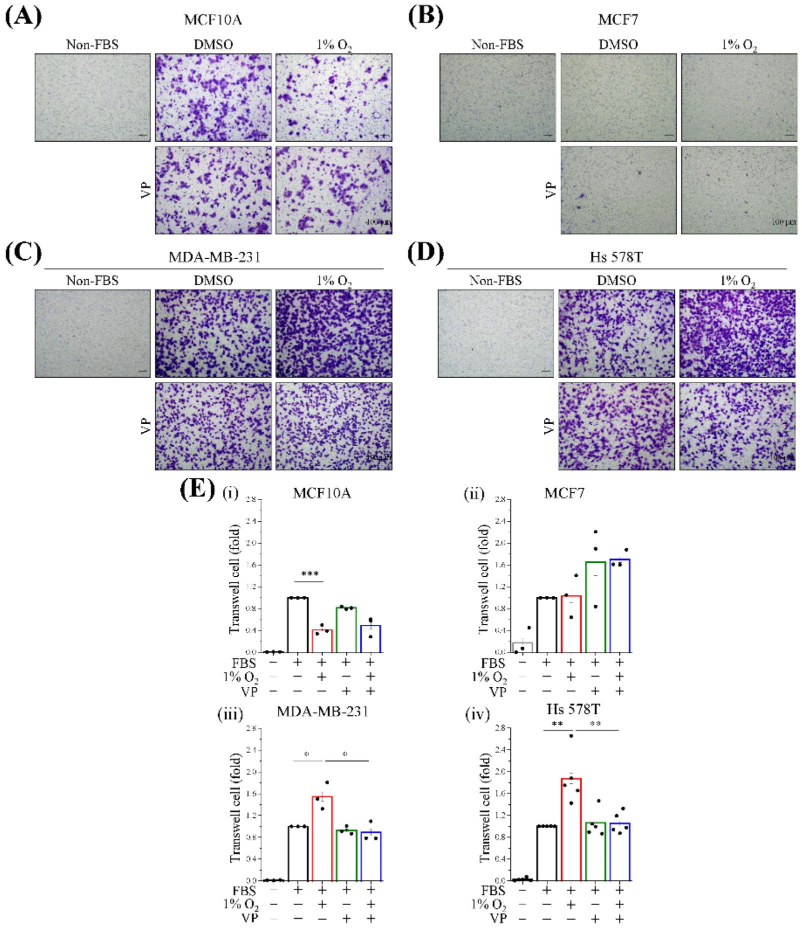

FIGURE S4 Hypoxia-induced YAP activation contributes to mesenchymal TNBC cells migration. Hypoxia chamber (1% O_2) was used to further illustrate that hypoxia plays a crucial role on cell migration. (A–D) Representative microscopic images of cells migrated through the polycarbonate membrane with a pore size of 8 μm by crystal violet staining of (A) MCF10A, (B) MCF7, (C) MDA-MB-231 and (D) Hs 578T cells. Scale bars, 100 μm. (F) Quantitative analysis of the relative of cells transwell. Data are expressed as mean ± SEM values of at least three independent experiments. *$p < 0.05$, **$p < 0.01$, ***$p < 0.001$.

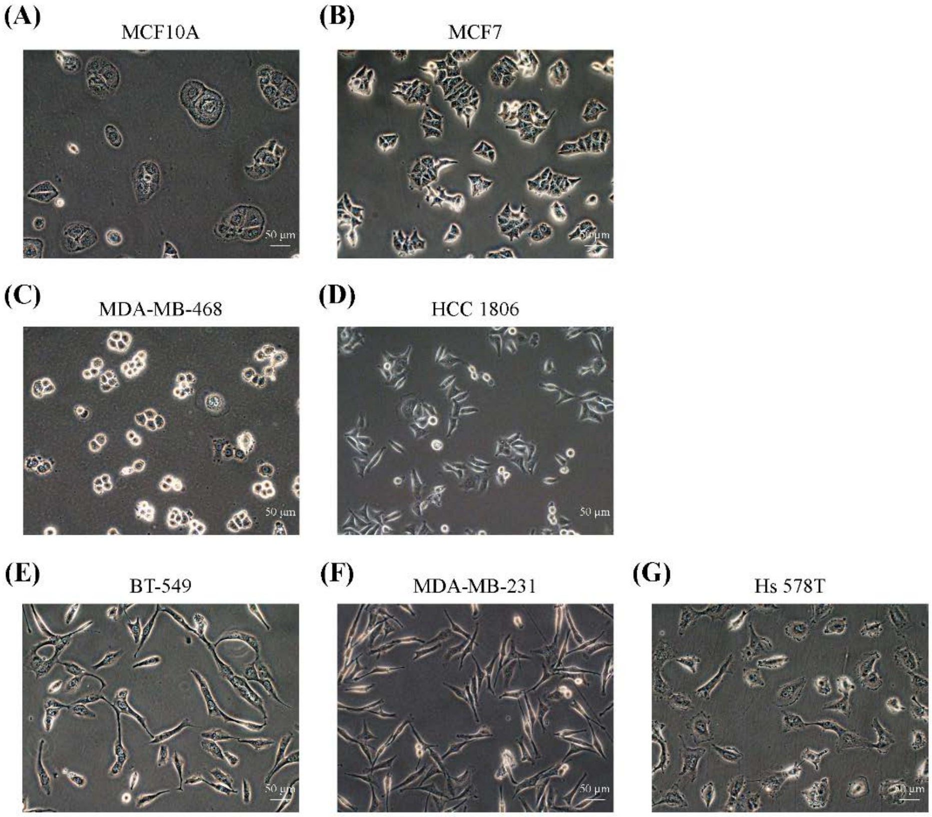

FIGURE S5 Morphologies of different cell lines. The cells were seeded and incubated for 24 h, then cell images were captured by bright-field microscopy. Representative images of (A) MCF10A, (B) MCF7, (C) MDA-MB-468, (D) HCC 1806, (E) MDA-MB-231, and (F) Hs 578T cells. Scale bars, 50 µm.

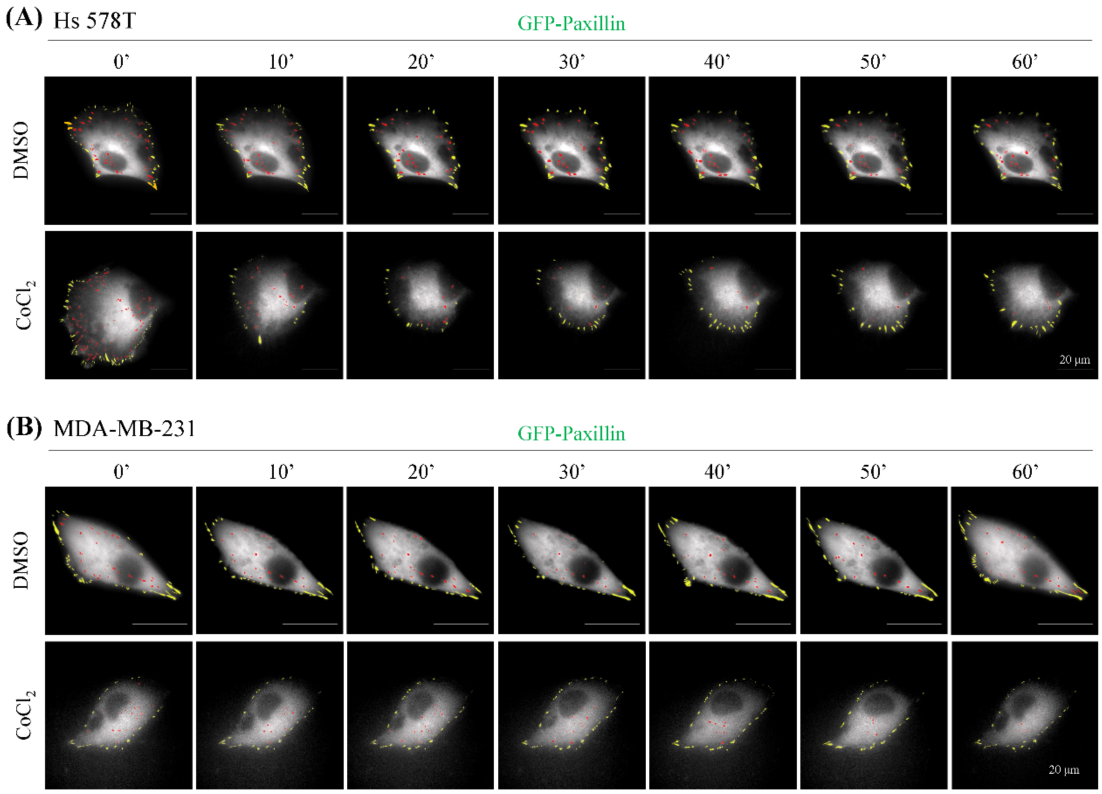

FIGURE S6 Dynamic of Paxillin under hypoxia conditions. The cells were seeded at high cell density, left for 24 h and were then transfected with GFP-Paxillin for 48 h. Following transfection, the cells were seeded and treated with or without 400 μM $CoCl_2$. Immunofluorescence images were obtained from time-lapse recording every 30 sec for 1 h by using TIRF microscopy. Representative images every 10 min of (A) Hs 578T and (B) MDA-MB-231 cells are shown. Scale bars, 20 μm. TIRF, total internal reflection fluorescence.
